# Opportunities and challenges in preventing violence against adolescent girls through gender transformative, whole-family support programming in Northeast Nigeria

**DOI:** 10.1186/s13031-022-00458-w

**Published:** 2022-05-12

**Authors:** Andrea Koris, Shadrack Steven, Veronica Akika, Cassondra Puls, Charles Okoro, David Bitrus, Ilana Seff, Julianne Deitch, Lindsay Stark

**Affiliations:** 1 Women’s Refugee Commission, 15 W. 37th St, New York, NY 10018 USA; 2 Mercy Corps Nigeria, 35 Patrick Bokkor Crescent, Jabi, Utako District, Abuja, Nigeria; 3 Brown School at Washington University in St. Louis, Campus Box 1196, 1 Brookings Drive, St. Louis, MO 63130 USA

**Keywords:** Adolescents, Humanitarian settings, Nigeria, Gender-based violence, Household violence, Gender norms

## Abstract

**Background:**

Household violence is one of the most prevalent forms of gender-based violence faced by adolescent girls in humanitarian settings. A growing evidence base demonstrates the extent to which multiple forms of familial violence, including intimate partner violence, violence against children, and sibling violence overlap in the same households. However, existing evidence of family support programming that effectively reduces violence against girls by addressing intersecting forms of household violence are limited, particularly in the Global South. Through a qualitative implementation evaluation informed by a grounded theoretical approach, we explored the perceived impact of a gender transformative, whole-family support intervention aimed at building adolescent girls’ protective assets against violence, among program participants in two communities of internally displaced people Maiduguri, Borno State, Northeast Nigeria.

**Methods:**

We conducted six in-depth interviews and six focus group discussions with adult caregivers; six participatory activities and four paired interviews with adolescent girls and boys; and 12 key informant interviews with program staff. Criterion sampling was used to recruit 21 male caregivers, 21 female caregivers, 23 adolescent boys, and 21 adolescent girls; purposive sampling was used to recruit 12 program staff to participate in qualitative research activities. We audio recorded, translated, and transcribed all interviews. In a collaborative coding process, a multi-stakeholder team used applied thematic analysis in Dedoose to identify emergent themes in the data.

**Results:**

Participants reported a decreased tolerance for and perpetration of violence against girls at the household level, and endorsed their right to protection from violence at the community level. However, alongside these self-reported changes in attitude and behavior, aspects of normative, patriarchal norms governing the treatment of adolescent girls were maintained by participants.

**Conclusions:**

This study builds the evidence base for gender transformative, whole-family support programming and its impact on preventing violence against adolescent girls in humanitarian emergencies. Situating our findings in a feminist analysis of violence, this study calls attention to the complexity of gender norms change programming amongst families in conflict-affected settings, and highlights the need for programming which holistically addresses the relational, community, and structural drivers of violence against girls in emergencies.

## Introduction

**Fig. 1:**
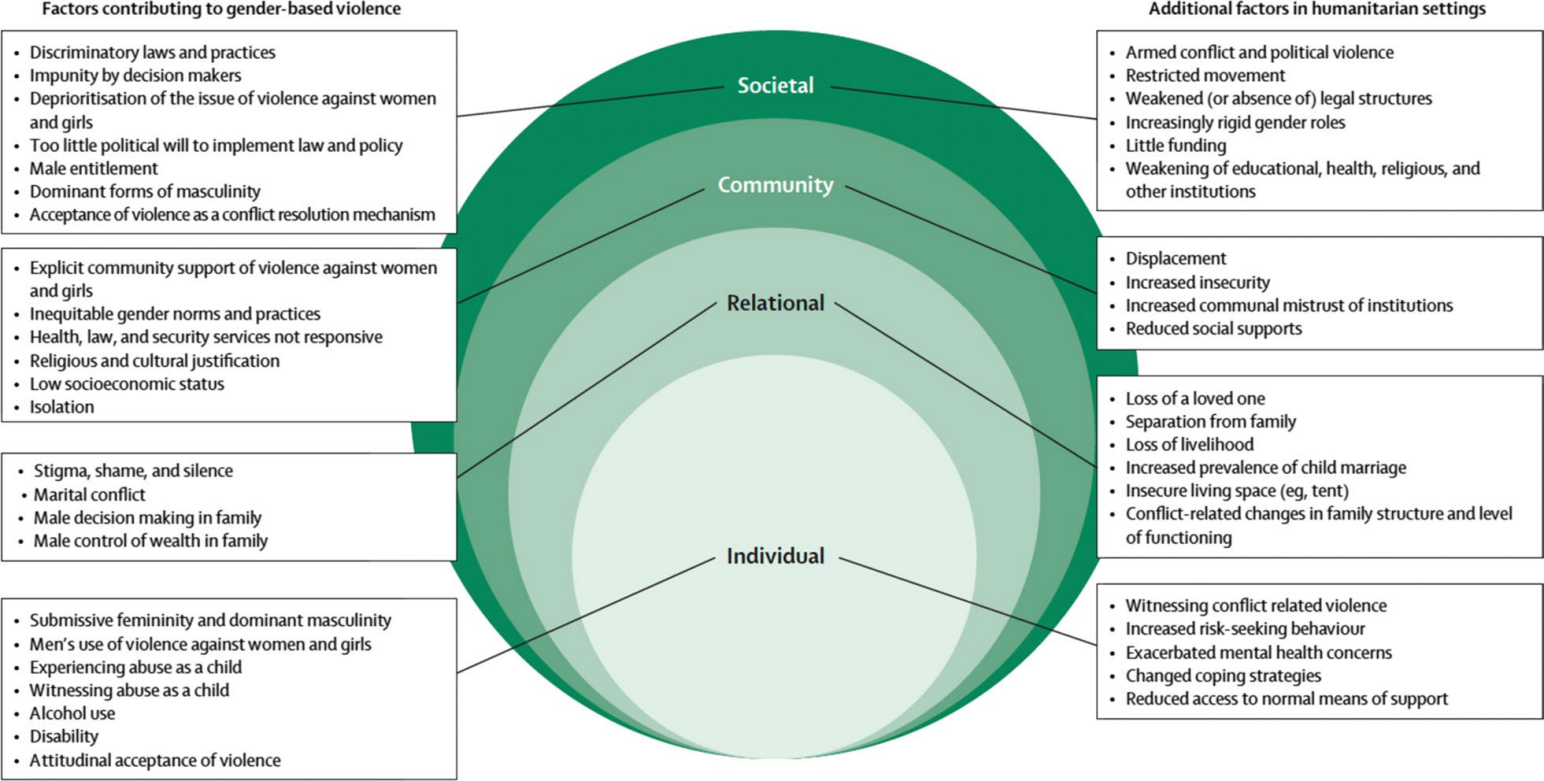
Socioecological determinants of gender-based violence in humanitarian settings. Note: Socioecological determinants of gender-based violence in humanitarian settings. Reprinted from “Gender-based violence against adolescent girls in humanitarian settings: a review of the evidence,” by Stark, L., Seff, I., and Reis, C. 2021., The Lancet Child & Adolescent Health, 5(3), 210–222. Reprinted with permission

Violence against women and girls, defined as acts of physical or emotional gender-based violence (GBV), is an intractable global issue driven by historically oppressive power relations that uphold male dominance and prevent the full advancement of women in public and private life [1]. While global estimates show that nearly one third of ever-partnered women and girls ages 15–49 have experienced physical and/or sexual violence in their lifetime [2], the threat of GBV is elevated in humanitarian contexts [3, 4]. Adolescent girls face increased risk; an extensive literature base attests to the heterogenous perpetrators and pathways of GBV they face, due to intersecting vulnerabilities related to age, gender, and additional risk factors associated with emergencies or displacement [5–7]. This matrix of vulnerability is best understood within an ecological framework, which suggests that violence is a function of multifarious factors that interact at different levels of the social ecology [5] (Fig. 1).

Over the last decade, a growing evidence base suggests that most acts of GBV perpetrated in humanitarian settings occur at the household and community level [8]. Humanitarian emergencies can intensify conflict within families; economic losses put strain on households, and rapidly changing gender norms triggered by displacement and other stressors can initiate or exacerbate existing cycles of domestic violence [6, 9–11]. Global evidence from both non-humanitarian and humanitarian settings confirms that intimate partner violence (IPV) and violence against children (VAC) by their caregivers frequently co-occur in the same households [12–15]. Additionally, while substantially under researched in low- and middle-income countries, several studies from high income settings suggest that sibling violence also co-occurs with IPV and VAC, and further contributes to cycles of polyvictimization within households [16–18]. Violence between siblings is mediated by verbal conflict [19], is associated with future violence victimization by peers [20], and can disproportionately affect adolescent girls [21].

Shared drivers of IPV, VAC, and sibling violence at the household level include conflict exposure, alcohol and drug use, income/economic status, mental health/coping strategies [6, 22], and social norms such as normalization of violent discipline, conceptions of masculinity based on aggression and control, and gender inequality [13]. These intersecting drivers can profoundly impact adolescent girls; in particular, girls living in households characterized by male dominance and patriarchal decision-making face elevated risks of violence [23]. Recent feminist analyses investigate how patriarchal norms act as a cross-cutting risk, influencing the co-occurrence of IPV, VAC, sibling violence, and violence against girls within households [22, 24, 25]. In a conceptual model of intersecting family violence, Namy et al. explore how patriarchal power is constructed and sustained via the family system: rigid sex/age hierarchies place men in a superior position to women, girls, and children; gender and childhood norms maintain this structure and consequently de-value women, girls, and children; and the use of violence to enforce norms by placing ‘dominant’ household members over ‘subordinate’ household members legitimizes various forms of violence [24]. Situated within the broader context of the patriarchal family structure, women’s use of violence against children, and siblings’ use of violence against each other, reveal attempts to exercise power over others, when unable to express power in other domains of family life. Within this framework, adolescent girls are placed at greatest risk of experiencing violence due to intersecting vulnerabilities related to their gender and age which play out across hierarchies within their families, intimate partnerships, and broader social networks [7].

It is well-documented that violence is learned, internalized, and reinforced; one of the strongest predictors of young people perpetrating or being a victim of GBV is if, while a child, they witness violence against a female caregiver in their household [26–28]. Recently, humanitarian and development organizations began addressing the cycle of violence against girls by focusing on building their ‘protective assets’. Broadly defined, protective assets are human, social, economic, or cognitive capital that are protective against violence, and support girls in navigating various risks as they progress through adolescence into adulthood [29]. Protective asset programs try to bolster known protective factors against violence, which may include individual-level factors such as building girls’ self-esteem, household-level factors such as augmenting caregiver coping strategies and strengthening the gender equity and functioning within girls’ immediate households, or community-level factors such as increasing the number of safe spaces or quality of response services [30–34]. Gender transformative family support programs are one such example, as they offer promising opportunities to build the protective assets of girls by targeting immediate cycles of household violence, while simultaneously reducing longer-term cognitive effects of trauma that fuel future violence victimization and perpetration [35–37].

However, evidence on gender transformative family support programming in humanitarian settings is limited, and empirical evaluations suggest mixed outcomes for reducing violence against adolescent girls at the household and community level [38]. An evaluation of “Parenting for Lifelong Health: Sinovuyo Teen”, a caregiver support program implemented in South Africa in 2012, found a significant effect of reduced physical and emotional abuse reported by caregivers and adolescents at one-month post-intervention, but at 5–9 months post-intervention, found no significant intervention effect on the same measure per adolescents’ self-report [39]. More recently, an evaluation of the COMPASS Program (Creating Opportunities through Mentorship, Parental Involvement, and Safe Spaces) implemented in Ethiopia, as well as the Democratic Republic of Congo (DRC) and Pakistan in 2016 found mixed results; in Ethiopia, there were improvements in social safety networks and attitudes surrounding gender, but no change in incidence of sexual violence [40].

### The Sibling Support for Adolescents in Emergencies (SSAGE) Program

The Sibling Support for Adolescents in Emergencies (SSAGE) program was designed to challenge intergenerational cycles of violence and prevent future perpetration of violence against girls through a whole-family support approach. The program was implemented by Mercy Corps Nigeria (MCN) from September–December 2020 in Bullabulin and Bayan Kwatas, two communities of internally displaced people (IDPs) within Maiduguri, the capital of Borno State. The SSAGE program used a gender transformative pedagogy following the Align Platform framework [41], with the goal of interrogating normative beliefs and interpersonal dynamics that fuel violence against girls and women, advancing equitable attitudes and behaviours within families, and encouraging positive masculinities. The whole-family approach was operationalized by enrolling adolescent girls, their male siblings, and their male and female caregivers into the program; facilitating synchronized interactive age and gender-specific sessions guided by gender transformative pedagogy; and encouraging intra-familial discussion on weekly topics throughout the duration of the program. The SSAGE curricula were adapted from MCN’s existing Life Skills curriculum, through a community-based participatory design process with IDP community representatives that ensured the curricula relevant and culturally appropriate. Four distinct but corresponding curricula were developed, and sessions were designed to be engaging and interactive, with a focus on stimulating self-reflection and discussion amongst participants on topics related to gender, power, violence, interpersonal communication, and healthy relationships (see Table 1: SSAGE curricula topics by participant group).

**Table 1:** SSAGE curricula topics by participant group

#	Adolescent girls	Adolescent boys	Female caregivers	Male caregivers
1	This is me!	What does it mean to be a man?	This is me!	What does it mean to be a man?
2	*What does it mean to be a girl? (Part 1) * How we learn to be girls (and boys) Sex and gender	*Gender socialization* How do we learn to be boys and girls	*What does it mean to be a woman? (Part 1)* Gendered values Sex and gender	*Gender socialization* How we learn to be men and women Sex and gender
3	*What does it mean to be a girl? (Part 2)* Gendered values Work we do and the value it’s given	*Gender roles* Gendered values Work we do and the value it’s given	*What does it mean to be a woman? (Part 2)* How we learn to be women (and men) Work we do and the value it’s given	*Gender roles* Gendered values Work we do and the value it’s given
4	*Power and empowerment* Power balance Discrimination	*Power and discrimination* Power balance Discrimination	*Power* Power balance Exploring the meaning of power Who has power and how do they use it? Discrimination	*Power and discrimination* Power balance Discrimination
5	*What is violence?* Types of violence Consequences of GBV Perpetrators of GBV	*Types of violence* Boy’s games Violence in our lives	*What is violence?* Types of violence The cycle of domestic violence Consequences of violence	*Types of violence* Boy’s games Violence in our lives
6	*Keeping safe from violence* Identifying violence My safety network	*Gender-based violence* Who uses violence and why? Consequences of GBV Cycle of domestic violence	*Keeping safe from violence* Violence in daily life Keeping safe from violence	*Gender-based violence* Who uses violence and why? Consequences of GBV Cycle of domestic violence
7	*My body, my rights* Physical and emotional changes in boys and girls Our rights Reproductive myths	*Preventing GBV* Violence in daily life Taking a stand against violence Power and violence	*Healthy couple relationships* My relationships Healthy relationships Healthy and unhealthy partners	*Preventing GBV* Violence in daily life Taking a stand against violence Power and violence
8	*Use and abuse of alcohol and other substances* What do we know about drugs? Drugs in our lives and communities	*Use and abuse of alcohol and other substances* What do we know about drugs? Drugs in our lives and communities	*Adolescence* Physical and emotional changes in adolescence Reproductive myths Adolescent girls’ rights	*Healthy couple relationships* Healthy and unhealthy partner relationships Consent
9	*Healthy relationships* My relationships Healthy and unhealthy relationships Family relationships	*Healthy relationships* Love and romance Consent Healthy and unhealthy relationships	*Adolescent girls and GBV* Understanding violence against AGs Consequences of violence against AGs = Protecting AGs from violence	*Adolescence* Physical & emotional changes in boys & girls Father-son relationships Adolescent girls’ rights
10	*Interpersonal communication* Listening skills Being assertive Resolving disagreements	*Interpersonal communication* Listening skills Being assertive Resolving disagreements	*Listening and communication* Listening skills Mother and child communication Empathy between mothers and children	*Protecting adolescent girls from GBV* Understanding violence against AGs Consequences of violence against AGs Protecting AGs from violence
11	*Decision making* Personal decision-making Resisting influences/ following through	*Decision making* Personal decision-making Resisting influences/ following through	*Healthy family environment* Family rules and expectations Decisions in the home Resolving disagreements	*Interpersonal communication* Listening skills Empathy between fathers and children Resolving disagreements
12	*Looking forward* This is me (part 2)	*Looking forward* What does it really mean to be a man? (part 2)	*Positive parenting* My parents’ legacy Positive parenting techniques	*Healthy family environment* Family rules and expectations Decisions in the home Positive parenting techniques
13	–	–	*Looking forward* This is me (part 2)	*Looking forward* What does it really mean to be a man? (part 2)

### Rationale and aims

Critical gaps remain in understanding the intersections of IPV, VAC, and sibling violence, particularly in regard to its impact on violence perpetrated against adolescent girls at the household and community level. In addition, existing evidence is skewed towards high-income settings, where structural factors and social norms that uphold patriarchal family structures likely differ from low-income settings. Research that centres the voices of adolescents living in contexts of humanitarian emergency in low-income settings are needed.

This paper seeks to address these gaps through a qualitative evaluation of a whole family support program in northeast Nigeria, focusing on two areas of inquiry: what are the changes in participant attitudes and behaviours related to violence at the household level? And what is the impact on adolescent girls’ protective assets against violence within their families and communities? This paper aims to contribute to conceptual and empirical understanding of the impact of gender transformative, whole family support programming on girls’ risk of GBV in humanitarian contexts, and to inform future prevention programming.

## Methods

### Study site

In northeast Nigeria, ongoing violence from the Boko Haram insurgency has generated wide-scale displacement across Borno State. In 2020, Maiduguri hosted over 2.1 million IDPs [42]. GBV is a significant factor of the conflict; high levels of abductions, rape, trafficking, forced marriage and forced religious conversions are associated with the ongoing conflict, which Boko Haram wields to finance their activities and inspire fear in communities that defy their ideological visions for society [43, 44]. Despite this reality, women and girls’ responses to violence in this setting are not monolithic. Amidst shifting terrains of risk, food insecurity, and severe poverty, some women and girls have seen in Boko Haram opportunities to advance their freedom and protection, and joined the insurgency as messengers, smugglers, recruiters, or religious converts [45]. Given the diverse ways in which women in northeast Nigeria engage with the ongoing social and political conflict, they may be cast as sympathetic or complicit with Boko Haram by Nigerian state forces or community members, and be victimized as a result. A recent study found that among a sample of 4,868 adult women from across northeast Nigeria, including Borno State, 33% reported experiencing sexual violence; perpetrators included Boko Haram insurgents, members of police or other security forces, unknown individuals within the community, and intimate partners [46].

Along with conflict-driven increases in the risk and incidence of GBV, existing patriarchal social-cultural norms which concentrate individual, community, and institutional power in the hands of men negatively affect women, children, and girls in northeast Nigeria. A mix of customary law, Salafi-inspired Islamic law, and modern statutory law create a set of social and economic relations that grant men primary rights to traditional titles, land ownership, and control of family resources and decision-making [47]. Traditional practices of bride price and child marriage that uphold male dominance and adversely affect adolescent girls are also prevalent in northeast Nigeria, including Borno State in particular [48]. Attitudes justifying the use of IPV in some situations are also widely held among both women and men across Nigeria, [49] as is social acceptance of using corporal punishment to discipline children [50]. Rates of IPV and VAC are high: a 2016 study found that nearly 37.9% of women reported experiencing severe combined physical, sexual and emotional abuse by intimate partners [51], and the 2014 Nigeria Violence against Children Survey found that approximately 50% of children reported experiencing physical violence in childhood by caregivers. Twenty-six percent of girls reported experiencing sexual violence [48]. Numerous Nigerian feminist scholars and activists have advocated for increased gender equality in Nigerian society [52–54] and while in 2015 the Nigerian National Assembly legally prohibited VAWG through passage of the Violence Against Person’s Prohibition Act, many states including Borno have not yet ratified the law [55].

### Implementation

Families with a female and male caregiver, as well as adolescent girls ages 10–14 and older male siblings ages 15–19 were eligible to participate. These criteria were determined to ensure that family members who typically hold power or decision-making and disciplinary authority over girls were included in the program. A team from Mercy Corps Nigeria mapped communities in Bayan Kwatas and Bullabulin to identify and invite eligible households. A total of 120 families and 480 participants were enrolled. The program was facilitated by four male and four female mentors, who were identified by MCN staff as respected and dynamic community members. Mentors underwent a week-long intensive training on gender transformative pedagogy, SSAGE curricula topics, and techniques for engaging participants in self-reflection. During program implementation, MCN staff met with mentors once per week to provide continued support and training. Mentors led separate sessions for adolescent girls, male siblings, and male and female caregivers in a synchronized, concurrent manner.

### Study design

We conducted an implementation evaluation guided by a grounded theoretical approach to qualitatively analyse the impact of the SSAGE program. Qualitative data were collected one month after the completion of program implementation. In total, 86 individuals were selected from the broader group of program participants, using criterion sampling based on factors such as location; gender; age; and program attendance rate in order to create a diverse sample and avoid bias in recruiting. Participants who were selected were not necessarily from the same family units. Participants were recruited to take part in the qualitative research activities, which included in-depth interviews (IDIs) and focus group discussions (FGDs) with caregivers, and paired interviews (PIs) and participatory research activities (PARs) with adolescents. Caregiver activities (IDIs and FGDs) were facilitated using semi-structured guides, which included open-ended prompts. Adolescent activities (PIs and PARs) were facilitated using youth-friendly approaches such as story completion and arts-based prompts, to foster increased comfort and engagement among participating adolescents [56]. All research activities were structured in part around vignettes depicting various scenarios related to relational violence. Participants were asked to discuss the vignettes from the perspective the fictional characters, and share their reflections and suggestions. Twelve SSAGE program staff were also purposively recruited for key informant interviews (KIIs), which focused on their perceptions of the feasibility, acceptability, and impact of program curricula and gender transformative pedagogical methods (Table 2).

**Table 2:** Qualitative methods and sample

Activity	Sample Size	Number of Activities Conducted	Topic Guide Focus
In-depth interviews	3 female caregivers; 3 male caregivers (6 total)	3 IDIs with female caregivers 3 IDIs with male caregivers	(1) Individual attitudes related to gender equality, and violence against women and girls (2) Individual perceptions related to change in household dynamics and familial relationships as a result of the intervention (3) Individual perceptions about the SSAGE program content and implementation
Paired interviews	4 adolescent girls; 4 adolescent boys (8 total)	2 PIs, comprised of two adolescent girls each 2 PIs, comprised of two adolescent boys each	
Focus group discussions	18 female caregivers; 18 male caregivers (36 total)	3 FGDs, comprised of 6 female caregivers each 3 FGDs, comprised of 6 male caregivers each	(1) Group perceptions of social norms related to gender equality, violence against women and girls, and gender identity (2) Group perceptions about the SSAGE program content and implementation
Participatory activities	17 adolescent girls, 19 adolescent boys (36 total)	3 PARs, comprised of 5, 5, and 7 adolescent girls respectively 3 PARs, comprised of 8, 6, and 5 adolescent boys respectively	
Key informant interviews	12 staff members involved with implementation of SSAGE	12 KIIs, comprised of 8 program mentors and 4 program staff	(1) Perceptions on the feasibility, acceptability, and impact of program curricula and gender transformative pedagogical methods
Total	98		

### Data collection

The WRC and MCN research team recruited 3 male and 2 female research assistants experienced in community protection programming and qualitative research who were unaffiliated with MCN. Research assistants were conversant in languages spoken in Borno State and conducted qualitative research activities in Hausa, Kanuri, or a mix of both following the preferences of the participants. Due to COVID-19 related challenges, data collection trainings with the research assistants were conducted remotely. Data collection monitoring was also conducted remotely. For all group activities, two research assistants were present; one responsible for facilitation and direct engagement with participants, and one responsible for observation and note-taking. Research assistants listened to the audio-recordings in the language in which they were performed, transcribed the audio directly into English, and redacted identifying information. Another Hausa-speaking MCN staff member not involved in the facilitation, translation or transcription of the interviews reviewed translated transcripts against audio files, to ensure accuracy of translation.

### Analysis

We employed a collaborative qualitative analysis approach [57] to the study data. Upon completion of data collection and transcription, a multi-stakeholder analysis team from MCN, WRC, and Washington University in St. Louis (WUSTL) participated in a virtual collaborative coding workshop. Prior to the workshop the team members reviewed a sub selection of the data, and used open coding to begin applying deductive categories and identifying inductive concepts in the data. During the workshop the team mapped out connections between categories and concepts using Google Jamboard, a collaborative workspace platform, and agreed upon a list of axial codes that represented major patterns in the data. Lead coders developed a preliminary codebook, which the analysis team piloted on a subsection of data using the constant comparison method [58]. The team reconvened for a second collaborative workshop, discussed feedback, and incorporated modifications into a finalized codebook.

In the final stage of analysis, the lead coders and the analysis team used the finalized codebook to co-code sub sections of the data in Dedoose. A The lead coders then created data displays to visualize major and minor themes in the data, and drafted thematic memos which explored the nuances and differences in themes by participant type, gender, and age cohort. Relevant quotations were incorporated into each memo to keep the analysis grounded in participants’ own words.

All study procedures were approved by the WUSTL institutional review board. All participants provided written, informed consent and assent. Study activities took place outdoors in MCN Safe Spaces which had auditory privacy, and adhered to COVID-19 safety protocols. Study procedures included a participant information sheet which provided study participants with anonymous reporting channels; and including channels for voluntary referral to specialized psychosocial support and protection services from MCN and partner organizations in case of possible emotional distress or protection risks associated with research study activities.

## Results

Our data elucidate participants’ perceptions of the SSAGE program’s impact on protective assets for girls. While participants reported changes in their attitudes and behaviors related to VAWG at the family level, collectively endorsed girls’ rights, and expressed a desire to bolster community protection of girls, our data also reveal circumstances where normative, patriarchal frameworks structuring the treatment of adolescent girls persisted. Overarching themes that emerged in the course of analysis are organized under the two areas of inquiry for this study: (1) attitudes and behaviours related to violence against girls within households; and (2) attitudes and beliefs about girls’ rights and protection. Subthemes within each area of inquiry emerged through a grounded theory analytic approach.

### Attitudes and behaviours related to violence against girls within households

The firm aim of this research was to understand the program impact on participant attitudes and behaviours towards violence against girls at the household level. While SSAGE participants reported greater understanding of gendered power differentials, improved communication amongst family members, and decreased perpetration of violence within households, aspects of familial hierarchies that uphold male dominance were maintained.

1. Power and communication

Participants’ reflection on power differentials across household relationships impacted the quality of communication amongst caregivers, between caregivers and adolescents, and with siblings. In particular, male caregivers’ reflection on their power relative to their family members seemed to impact their communication style. One mentor shared a key message from the program that they felt resonated deeply with male caregivers: “If a child brings a conversation to you, listen to him. Pay attention and provide affection so that you find what is wrong with him. Don’t shout at him, don’t harass him, be simple with him so tomorrow even if something happens he will be able to share it with you.” In describing perceived changes to the styles of communication used with their family members, male caregivers began to collectively express alternative masculinities embodying more egalitarian forms of manhood: “Back then we used to yell when they rushed to us when they saw us coming back home. But now we realized that was not right, we need show our children love and respect if we want them to be good in society.”

Changes to male caregivers’ communication style seemed to have broad-reaching impacts across households. Female caregivers felt that changes in their spouses’ communication style enabled shifts towards more egalitarian relationships within the family: “You see, before we didn’t sit together to make decisions, but now we do. And he also seeks my advice, too. Sometimes if I am unhappy, he wants to know what’s disturbing me, and he also does the same to his children.” Another female caregiver discussed how her husband’s increased attentiveness to his children led to his allocating extra resources for their daughter’s medical care:

So, you see, even my husband has changed a lot. Before when my children were sick, I would go and look for money to buy drugs and take care of them. With all girls and only one boy, he usually didn’t bother with their wellbeing, but everything has changed based on what he was taught in this program. Now, he asks about their wellbeing, and if they are sick, he will bring a doctor to the house to treat them. Truthfully, I am really happy. My husband now asks about our wellbeing, and that makes me happy.

Adolescent boys were also impacted by the changes in attitude and behaviour of their male caregivers. Some felt more comfortable sharing their own thoughts and emotions with their fathers: “A son can now speak with his father freely and confidently, and the father understands his children’s views and ideas…all because of this program.” Changes in the way their fathers communicated with them prompted others to reflect on how they engaged with their sisters. One adolescent boy shared, “I used to not smile at her because I thought she would underestimate me, or look down on me if I did. But I now realize that with all these angry faces, being unfriendly will never solve any problem between us. Another talked about modelling the communication style of his male caregiver when managing conflict with his younger sister: “Like before, if I talked to her, she would insult me. And when she did something like that to me, I would insult her. But now that we started coming to this program, we gained a lot from it. Now, even if she did something wrong to me, I just leave her or I tell her so she will understand and stop doing it.”

(2) Changes in perpetration of household violence

In addition to changes in communication, participants reported decreased perpetration of violence across household relationships, following engagement with the SSAGE program. Many of the participating adolescent boys reflected on their use of violence and negative power to coerce their sisters into conforming to certain behaviours, or adhering to their requests, sharing stories about how they “used to fight, my sister and I, because I used to make her do things for me at home…. Even though I can do things by myself, I used to make her do it for me just because I didn’t want to do hard work.” SSAGE sessions such as “Power and Discrimination” and “Gender-Based Violence” impacted the boys, by fostering an environment for them to evaluate the root causes and repercussions of their use of violence against their sisters. One participant shared: “I used to force her to wash all my clothes, and I would send her to buy me soap even though she had places to go. I would forcefully stop her from going out, but after this Mercy Corps program I realized that all what I was doing was wrong.” Another participant reflected about how his previous use of violence against his sister negatively impacted their relationship: “Back then my relationship with my sister was not good because I used to shout and yell at her, and we quarrelled a lot. But when we started attending this program, we are now living peacefully and in harmony with one another. I have stopped pushing her against the wall to force her to do things for me.”

Both caregivers and adolescents reported changes in the normalization of violence against children within families. First, female caregivers overwhelmingly shared their change in opinion regarding the use of corporal punishment with their children: “Well! I learned many things in this program, for example my relationship with my children. Before I started coming to this program, I was beating my children. But after I started participating in this program, I have corrected my mistake.” Adolescents corroborated this change, remarking on the shift they observed in their caregivers’ behaviour towards them and their siblings: “They [caregivers] are learning a lot of things. When they reach home and see children fighting, they instead separate them and discipline them rightfully. And that will put us on the right path.”

In lieu of using violence to reinforce disciplinary lessons and require particular forms of behaviour from their children, many female caregivers discussed tactics highlighted in the SSAGE program including establishing open communication and active listening. These tactics of ‘good’ discipline were often framed in aggregate as ‘enlightening’ children; drawing children into discussion and establishing mutual understanding of the importance of particular behaviours for the wellbeing of the child, and the household. Participants in one focus group shared, “You can call your child and encourage him or her. Beating and insulting will never make your child good. When you enlighten your children, they will be interested to go and learn more…”.

Male and female caregivers both acknowledged a link between parental treatment of children, and children’s treatment of siblings or others in the broader community. “We discussed things like how to raise a child to be a good man, because beating a child won’t help most of the times.” Participants from another focus group expressed the importance of using nonviolent discipline with “…not just the adolescent girls, but the adolescent boys, too. Not only discipline them, but discipline them in a good way so that they can be good children in the community.”

Lastly, female participants spoke candidly about forms of intimate partner violence commonly experienced in spousal relationships across their community, including physical and emotional violence, substance abuse, and male dominance over familial resources. Many felt that the SSAGE program empowered them with knowledge to better understand risk patterns in their spouses’ behaviours, and provided them with tools to mitigate conflict. For example, they reported that the SSAGE sessions which fostered reflection about the intersection of drug/alcohol use and male violence impacted their spouses’ behaviours and reduced perpetration of IPV in the short term: “And our husbands too have really changed. Some husbands will go out and if they come back home, they won’t smile, sometimes you won’t even know when your husband goes out and when he gets angry outside, he will come home and pour the anger on you. But all this has changed when the program started.” Male caregivers also reflected on how poverty, alcohol, and drug use can interact with power differentials between husband/wife and parent/children to exacerbate violence in the family. Several men shared their realizations about the impact of their own drug use—which was often expressed as a coping mechanism for dealing with the many structural challenges faced by displaced communities—on their family. “Before when I got upset, I would normally take drugs to cool myself, which made me misbehave and led me to do bad things…. Anyone that is not in their senses can do anything. But in the end, I understand when you use something it won’t only affect your own wellbeing. From then, I stopped it, and I understand it is bad.”

Some of the participatory activities meant to spark self-awareness and empathy impacted male caregivers, causing them to self-reflect on their use of power and violence against their spouses. This mentor shared his observations on the profound impact of a ‘Push and Pull’ activity, which he facilitated with male caregivers:

So, the two participants that were involved in the activity that had to do with push and pull. After the session I invited them to share what they felt about the role play. So, the man in the role play who was pushing, said it made him realize how he, how men, when they push someone, they feel more powerful than the other person, and sometimes they just need it to feel happy. So, when I asked the other person, the one that was pushed, he said he can just imagine how his wife has always felt whenever he pushes her around, you know, whenever he acts superior over her. So, the role play is very practical and it sends the message so that it is not easy to forget. Another participant, for instance, has said that he wants to do this with his wife, that going forward, he will remember this roleplay, and he will be like, ‘Okay when you do this, it does not make you happy, because when someone did it to me during the session, I was not happy about it.

(3) Maintenance of household hierarchies that uphold male dominance

While many participants perceived shifts towards more egalitarian and less violent relationships following the SSAGE program, the data also show instances where unequal power relations across gender and age hierarchies within households persisted. Several male caregivers felt that the functioning of their household had improved following the SSAGE program due to their children’s increased obedience and adherence to age/gender norms expected of children (particularly, female children): “But when we started attending the program, the girls started coming to us and asking about what they taught us in our part. And we would tell them to stop misbehaving, and to follow our instructions and advice.”

Similarly, several adolescent boys described how the SSAGE program reminded their younger, or female siblings to treat them with respect and deference: “If they didn’t take advice from you before, but now since they started coming to this program they will understand and start taking your advice and whatever you tell them. And you should also listen to what they have to say.”

Some adolescent girls internalized this reinforced power relation. One participant shares her perspective on why her relationship with her older brother has improved since the SSAGE program: “For instance, my brother used to ask me to do something for him and I would refuse to do it, but after attending this program if he sends me to do something I will go quickly and do it.”

Lastly, in some group discussions, male caregivers infantilized women and shared an accepted belief in women’s intellectual inferiority. This male caregiver shared his perspective on the role he needed to play in ensuring his wife understood the SSAGE program messages:

Despite the fact that peoples’ understanding differs, some people understand at once. For other people, you will repeat it and they will still not understand, especially women. Women do not understand fast, while if you tell a man once or twice, he will understand. Your woman, even if you repeat it ten times, she will not understand what you mean. Because of that, if there is any problem between us [in the household], what I will do to settle the issue is tell her: “Remember that every week we used to attend the Mercy Corps program, where they lectured us…about good household living, and our neighbours?

On a few occasions, female caregivers reflected their internalization of this stereotype themselves. One female caregiver explained, “You know, us women have a dumb head; it is not everyone that God has given wisdom.”

### Attitudes and beliefs about girls’ rights and protection

A second aim of this research was to understand the program impact on household and community protection for adolescent girls. While SSAGE participants collectively endorsed adolescent girls’ rights and shared a desire to create community protective infrastructure, persisting aspects of patriarchal norms governing treatment of adolescent girls also emerged in the data.

1. Increased Commitment to Girls’ Rights

Female caregivers were impacted by the SSAGE program sessions on “Adolescent Girls and GBV” and “What is Violence?” Many felt that these sessions confirmed their own lived experience of violence, and further informed their understanding of the specific pathways of violence affecting adolescent girls that occur in their community. One focus group of female caregivers shared, “Everything that we were taught is the reality…like what we were taught about adolescent girls who are sent to the shop to buy some things, while the owner will be giving her things like sweets to get her attention to abuse her.”

Others felt the sessions informed their analyses of how harmful gender norms operational at the household level can increase girls’ risk of violence, such as how beliefs linking familial honour to girls’ sexual purity can influence parental responses to sexual assault. When describing the likely response of a caregiver if their daughter were to experience sexual violence in their community, one focus group participant shared: “…her parents will be quiet about it, so that the community people will not hear about it, so that they will not be disgraced.” Other participants in the focus group condemned the response of the caregivers, and pledged to “…defend and protect [girls’] rights against sexual exploitation and abuse.” Another female caregiver offered a particularly strong indictment of violence against girls, and commitment to justice for them:

As a woman, if your daughter is sexually abused, she’s not happy and the whole community is against her and putting all the blame on her. Instead, this case should be reported and justice should be done... these things do happen. Seeing the victim being seen as a bad person, people don’t want to associate with her, no man wants to marry her, and she’s scared of reporting the case so that she won’t be exposed. But it [shouldn't] be like that. Anyone who is caught sexually abusing a girl should be reported and punished, and justice should be done for the girl. When that is done, nobody would do that again.

(2) Endorsement of Household and Community Protective Infrastructure for Adolescent Girls

Caregivers acknowledged that dangerous terrains within their communities posed different threats to adolescent girls and boys, but many expressed feeling ill-equipped to manage the myriad risks posed to their children. A SSAGE program mentor described some of the barriers caregivers face in keeping their daughters safe from violence:

…parents don’t know where to find solutions to these problems which are very rampant. So, some people may abuse adolescent girls, especially those who have power with money, or the offer of marriage. They say, if you talk about this then I will kill you, or I will send somebody to lock you up somewhere. The girls and the caregivers have fear of this. So, the participants were very happy with this child protection session because the session focused on the consequences of violence, of rape, the disadvantages of early or forced marriage of children. All these things are the consequences of types of GBV. So, we enlightened them on the consequences, and they understand the reality better. So that’s why they felt that topic and activities were meaningful.

Adolescent boys also talked about their role in protecting adolescent girls, and reflected on how their current behaviours may help or hinder their sisters access justice in the event of violence or assault. Many shared that previously they were likely to fight other young boys who physically or verbally harassed their sisters, but now they were more comfortable identifying other avenues for conflict mediation that would centre the needs of their sisters, as opposed to their own needs for retaliation against their peers. “Before, if they insulted my sister in the community, I would gather my friends to go fight them and also create serious violence in the community. But as the result of this program, I understand that is not good. I would now go and report it to security, or to our district head and tell them to take decision.”

However, despite the SSAGE program’s focus on introducing different avenues for seeking justice for victims of GBV, participants reflected on how corruption and the lack of functional justice mechanisms at the community and state level contribute to a reality where perpetrators of GBV are treated with impunity and victims have limited legal recourse: “Pertaining to gender-based violence… We have been seeing it happening. The issue is normally taken to the police, and if the perpetrator has one thousand or two thousand naira he will bail himself out.”

Given the loss of traditional, community, and institutional protective infrastructure as a result of conflict-related forced displacement that many families within Bayan Kwatas and Bullabulin have experienced, caregivers expressed a need to recreate networks of community protection inclusive of adolescent girls. They framed community protection as mobilizing other adults and elders to provide advice, guidance, and watchful oversight to keep girls safe, and prevent boys from perpetrating violence. Their suggestions reflected a desire to reconstruct a social fabric of shared responsibility for the care of children. One male caregiver shared his belief in the importance of a cohesive community to help protect, and raise, children.

It is supposed that you should correct your own [children]. I also went to my friend’s shoulder to tell her that she should correct mine too because of that. Let us come together, all of us, to make the thing right. But if we leave it like that, for everybody to correct their own, we will not get the cooperation and peace. Because we have to come together to correct for things to be good. We are supposed to bring our children under us, to show them the proper things for them to do, and what is not proper for them not to do**.**

In light of the absence of protective infrastructure that many participants desired to recreate, male and female caregivers heavily emphasized the role of protective parenting in maintaining the safety of their adolescent girls. Discussions about ‘responsible’ and ‘irresponsible’ forms of parenting abounded in the data. Caregivers felt that both parents should be involved, sensitized to risk, and prepared to teach their adolescent girls to recognize situations where they may encounter violence or abuse in their external community:

Yeah, participation of both husband and wife is very important because some of the parents are so careless about their daughters. They were always proud that someone gave their daughters a phone for about 80 to 90 thousand without any reason, and she was not even engaged with anybody, but he bought her a phone. Truly, I am very glad to see that both husband and wife were also participating in this program.

This male caregiver shared his perspective on appropriate parenting tactics to reduce his children’s risk of violence:

Yes, there are things I learned…Like the time they are going to bed, and the time they will wake up. You see, monitoring activities of the children, if 10pm reached, even if I am not in that home, I will make sure I make a call to confirm if all the children are at home. I then make sure all of them are at home by that time, even if you want to travel or want to go for a place. I have to be notified that you will be going somewhere, or there is someone’s wedding you are going to attend.

(3) Emphasis on protection can limit girls’ mobility and freedom

Heightened protection of adolescent girls was seen as a necessary precaution by caregivers, commensurate to the level of risk present within the Bullabulin and Bayan Kwatas communities. Alongside these realities, however, the unintended consequences of increased protective parenting were apparent; caregivers advocated for amplified control over girls’ behaviours, a reduction in girls’ mobility throughout their community, and further restrictions of their freedoms. When discussing the importance of preventing their daughters from going out at night – a key message gleaned from the SSAGE program—male and female caregivers framed VAWG as a primarily female problem, which could be addressed through controlling their daughters’ behaviour, restricting their movements at night, and sensitizing them to risks they may face. These male caregivers shared the perception widely held amongst participants that “there are places a female should be and places she shouldn’t be, because not every place is good for a female to be.” Female caregivers in another focus group stressed the importance of restricting their daughters’ movements after nightfall: “For the adolescent girls, we were taught not to allow them to go out once it’s late in the evening from 6 pm to prevent incidences of rape. So, we all stopped them from going out when it’s late. Once it’s 6 pm, our adolescent girls do not go out.”

## Discussion

This research explores the impact of a gender transformative, whole-family support program on attitudes and behaviours related to violence against adolescent girls at the household level. While family support programs typically focus on strengthening caregiver practices [35, 40], the SSAGE program expands the scope to include the promotion of gender equitable attitudes and nonviolent interactions for the whole family, including male siblings. By engaging whole family units in synchronized, gender transformative curricula, the program addressed the unique ways in which attitudes and behaviours related to masculinities, femininities, and age shape individuals’ acceptance, perpetration, or experience of violence within households.

Participant-reported shifts toward more egalitarian familial relationships, increased endorsement of girls’ rights, and decreased violence perpetration suggest that gender transformative approaches may support the prevention of violence towards adolescent girls at the household level. In particular, caregiver feedback highlighted the pivotal role that practicing self-reflection, considering power differentials across family relationships, and modelling nonviolent communication played in initiating changes to relationships and pre-existing patterns of violence. Adolescent boys seemed to be strongly impacted by the shifts in their caregivers’ behaviour within the household, and mirrored nonviolent communication and engagement with their sisters. This finding is corroborated by a recent systematic review on risks and protective factors associated with family violence among refugee families, which finds that individual coping mechanisms involving reflection, nonviolent communication, or culturally-specific mindfulness techniques among caregivers can be protective against cycles of family violence [33]. SSAGE sessions which encouraged participants’ interactive, self-reflective engagement via participatory activities may have been particularly impactful in helping participants reflect on their use of power in relation to others, and build positive coping mechanisms for stress; this is reflected in various evaluations which attest to the efficacy of pedagogical tools employed in gender transformative programming [59–61]. Further impacts of the SSAGE program on participant family functioning are reported elsewhere (Seff et al., 2021 In Press).

Despite the narratives of personal change expressed by participants, our data also reveal moments of contestation, where patriarchal norms governing familial relations and attitudes related to girls’ safety from violence persisted. Perhaps most notable was caregivers’ endorsement of stricter parenting measures to better control their daughters’ behaviour and mobility, and their valorisation of girls’ obedience. Caregivers and adolescent boys felt that girls’ adherence to gender and age norms kept them safe from violence; conversely, girls’ transgression or ‘deviance’ [62] from norms related to their behaviour, dress, speech, affect, and mobility was viewed as a driver of male violence against them, both at home and in the broader community. This rationalization of violence against adolescent girls is explored at length in various feminist analyses of VAWG, as well as qualitative studies exploring how gender norms are reinforced through stigma and violence against girls in contexts such as humanitarian emergencies [63, 64]. While SSAGE caregivers increasingly endorsed girls’ equal rights to justice, they also felt that the SSAGE program improved their parenting practises, better enabling them to ensure their daughters’ compliance with various gender and age norms, and limit their mobility when deemed necessary.

Maintenance of patriarchal hierarchies within the household and increased parental control of girls did not align with the gender transformative aims of the program. One possible explanation for these findings is that these behaviours served as adaptive responses to prevent violence against girls in the short and medium term, given IDP families’ lived experiences of resource scarcity, poverty, and protracted violence in Maiduguri. Research on parenting practices in various conflict-affected settings corroborates these findings; recent studies from high income settings show that parents employ various strategies to control their adolescents’ mobility, relationships, and activities to prevent their potential exposure to community violence [65–67]. Other studies show that some parents elect to harshly punish adolescents as a way to eradicate behaviour they believe could lead to future exposure to violence [68]. Despite this rationale for adaptive parenting responses to prevent violence exposure, increased control over girls’ mobility and maintenance of patriarchal hierarchies within families may further contribute to oppressive power relations which restrict the freedoms of girls across multiple domains of their lives in the long term.

As with any strategy for effecting change, gender transformative approaches have limitations and an exploration of them may shed light on the complexity of findings from the SSAGE programme. Gender transformative approaches which focus on norms change aim to reshape gender relations at the individual, relational, community, or structural levels. However, a recent systematic review of rigorously evaluated programmes aimed at changing gender norms to improve health outcomes found that of 59 identified programmes, most focused on improving the individual agency of beneficiaries, and only 10% showed evidence of broader norms change [61]. Overemphasizing gender-norms change at the individual or relational level may limit opportunities for violence prevention, as it can unduly centre the agency of the individual in GBV prevention strategies. While gender norms-change programming can impact the beliefs, attitudes, and behaviours of individual perpetrators [69], it is perhaps equally urgent to design programming attentive to the overarching structural factors which shape individual agency and pattern community-level norms [70, 71]. Feedback from SSAGE participants and mentors suggests that the program impacted attitudes and behaviours related to gender equity and violence within families, but did not address broader community or structural-level factors that underpin the perpetration of GBV against adolescent girls in northeast Nigeria. The SSAGE theory of change and program model was not designed with components in mind to address supra-household levels of the ecological framework, and based on the data, this was found to be a shortcoming in the extent of the program’s effects on preventing violence and promoting gender-equitable behaviours. Future gender transformative family support programming should engage with other levels of the social ecology in order to reinforce environments which support and sustain the transformation of VAWG at the individual and relational levels [61, 72, 73]. While addressing certain structural factors that contribute to GBV (such as protracted violent conflict) is currently seen as outside the scope of most humanitarian programming, it is nonetheless critical that theories of change are developed alongside an acknowledgement of the role that factors such as war, resource scarcity, food insecurity and unstable housing play in shaping gender relations at the community, relational, and individual levels [74–76]. This may help contextualize complex program outcomes of family strengthening or violence prevention programming within a more holistic understanding of individual and collective social change processes.

This study was not without limitations. First, while the evaluation was qualitative in nature and possibly subject to certain biases, such studies have been shown to offer important insights into complex dynamics underpinning program effectiveness [77–79]. Second, although our topic guides did not intentionally restrict discussions to specific forms of violence, the adolescent-friendly methodology inherently focused the scope of discussion by prompting participants to consider specific story-based scenarios. For ethical reasons we did not ask direct questions related to individuals’ personal experience of violence perpetrated by family members, community members, or by parties to the Boko Haram insurgency. As a result, our findings do not include reference to non-partner sexual violence, female genital mutilation/cutting, or forced or early child marriage although existing data indicate these forms of violence are relatively prevalent for adolescent girls in northeast Nigeria [46, 80, 81]. A third limitation of this research was that data from adolescent girl participants was limited; this was likely due to challenges the research team encountered in remotely training data collectors on the use of adolescent-friendly, participatory methodologies with younger adolescents (age 14 and under). Research trainings were conducted remotely due to COVID-19 related travel restrictions.

## Conclusions

Findings from this study highlight the impact of the SSAGE program on attitudes and behaviours related to violence against women and girls, and they call attention to the complexity of gender norms change programming in contexts of protracted conflict and displacement. Despite some unintended participant interpretations of the curricula, the SSAGE program sparked meaningful consideration of and commitments to gender-equitable relationships and nonviolent interactions amongst participating families. Further evidence-based programming is urgently needed to strengthen family support networks as a protective asset for girls living in and around Borno State, given the complex ways in which they experience violence victimization both by known and unknown individuals. Additionally, quantitative research that measures the long-term impacts of gender transformative whole-family support programming on attitudes, norms and behaviours related to VAWG are required. The findings from this study offer insights that may be of relevance to future practitioners in humanitarian settings; in particular, program implementers should more deeply consider the interplay between relational/community level factors with structural factors, which may shape the construction of gender norms and the masculinities/femininities available to individuals in a given context.

## Data Availability

The data that support the findings of this study are available from the corresponding author, LS, upon reasonable request.
